# An Integrated Approach for Biofortification of Carotenoids in Cowpea for Human Nutrition and Health

**DOI:** 10.3390/plants13030412

**Published:** 2024-01-30

**Authors:** Kpedetin Ariel Frejus Sodedji, Achille Ephrem Assogbadjo, Bokyung Lee, Ho-Youn Kim

**Affiliations:** 1 Smart Farm Research Center, Korea Institute of Science and Technology (KIST), Gangneung 25451, Republic of Korea; frejusariel@gmail.com; 2 Division of Bio-Medical Science and Technology, KIST School, Korea University of Science and Technology (UST), Daejeon 34113, Republic of Korea; 3 Non-Timber Forest Products and Orphan Crop Species Unit, Laboratory of Applied Ecology (LEA), University of Abomey-Calavi (UAC), Cotonou 05 BP 1752, Benin; assogbadjo@gmail.com; 4 Department of Health Sciences, The Graduate School of Dong-A University, Busan 49315, Republic of Korea; 5 Department of Food Science and Nutrition, Dong-A University, Busan 49315, Republic of Korea

**Keywords:** biofortification, omics, cowpea, carotenoids, plant factory, speed breeding

## Abstract

Stress-resilient and highly nutritious legume crops can alleviate the burden of malnutrition and food security globally. Here, we focused on cowpea, a legume grain widely grown and consumed in regions at a high risk of micronutrient deficiencies, and we discussed the past and present research on carotenoid biosynthesis, highlighting different knowledge gaps and prospects for increasing this micronutrient in various edible parts of the crop. The literature survey revealed that, although carotenoids are important micronutrients for human health and nutrition, like in many other pulses, the potential of carotenoid biofortification in cowpea is still underexploited. We found that there is, to some extent, progress in the quantification of this micronutrient in cowpea; however, the diversity in content in the edible parts of the crop, namely, grains, pods, sprouts, and leaves, among the existing cowpea genetic resources was uncovered. Based on the description of the different factors that can influence carotenoid biosynthesis and accumulation in cowpea, we anticipated that an integrated use of omics in breeding coupled with mutagenesis and genetic engineering in a plant factory system would help to achieve a timely and efficient increase in carotenoid content in cowpea for use in the food systems in sub-Saharan Africa and South Asia.

## 1. Introduction

Micronutrient deficiencies are among the major causes of poor health and reduced economic development in the developing world [1]. The importance of stress-resilient and highly nutritious food crops in the current food systems context cannot be overemphasized. Legumes are a source of important secondary metabolites including carotenoids [2], and they play a significant role in food and diet diversification and ecosystem protection [3].

Carotenoids are the second-most abundant naturally occurring pigments on earth, synthesized by plants, which fulfill important physiological functions. Carotenoids in higher plants are found in photosynthetic tissues and non-photosynthetic tissues [4]. The main carotenoid pigments found in the photosystems of plants include α-Carotene and β-carotene, which are further hydroxylated to produce xanthophylls (e.g., lutein and zeaxanthin) [5]. The crucial roles of carotenoids and their metabolites in photooxidative protection and photosynthesis, not to mention nutrition, vision, and cellular differentiation, make them an important class of biological pigments [6]. In cowpea, carotenoids are mainly present in seeds, leaves, and pods, which contribute to the antioxidant properties of this legume [7].

Over the past decade, biofortification has gained recognition as a cost-effective, complementary, feasible means of supplying micronutrients to populations that may have limited access to diverse diets, supplements, or commercially fortified foods [8]. Biofortification uses agricultural practices and breeding as a public health intervention and, as a result, has the potential to more effectively reach the rural poor who are often the most affected by micronutrient deficiencies [1]. Although grain legumes are an integral part of the food systems in sub-Saharan Africa and South Asia, only limited efforts have been made to increase their nutrient contents [9].

The biofortification potential of grain legumes including cowpea remains underexploited [10]. Cowpea is one of the most important legume grain crops, mainly grown and consumed in sub-Saharan Africa and South Asia, regions at a high risk of vitamin-A deficiency [11,12,13,14]. The profiles of carotenoid content in cowpea grains are comparatively lower than the content in other legume grains, including lentils, red beans, and pigeonpea [15]. Most of the biofortification research in cowpea has been focused on iron and zinc [8,16,17], with no effort for increasing carotenoids. Therefore, the objectives of this review were to provide a critical and comprehensive update of the research on carotenoid biosynthesis and accumulation in cowpea, to identify the knowledge gaps as well the existing resources, and to discuss the prospects for carotenoid biofortification in cowpea for human health and nutrition.

## 2. Importance of Carotenoids for Human Nutrition and Health

**Figure 1:**
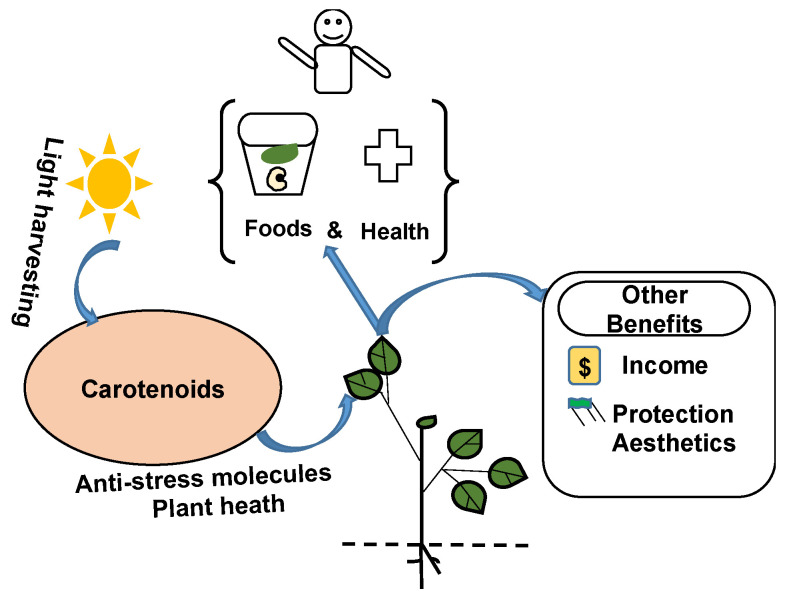
Carotenoids as multifunctional and multipurpose plant metabolites.

Carotenoids are micronutrients with essential functions and benefits to humankind (Figure 1). They contribute to harvesting light to plant chlorophyll for photosynthesis [18], thereby providing indirect sources of energy, nutrients, and clean air to humans. They exert functional roles in plant hormone synthesis and photoprotection and act as scavengers of reactive oxygen species, which enables plants to withstand stresses [19,20] and fully express their potential to provide diverse services and functions, including foods, health, protection, and income.

The importance of carotenoids for humans goes beyond nutrition. Carotenoids are health-promoting organic compounds. They contribute to the human antioxidant defense system and reduce the risks of cancer, eye, and age-related diseases [21,22,23]. Among other carotenoids, lutein is highly recognized for its anti-inflammatory properties; it helps to prevent macular disease, to improve cognitive function, and to reduce the risk of cardiovascular diseases [24]. Carotenoids, especially β-carotene, α-carotene, and β-cryptoxanthin, contain unmodified β-ionone groups that are precursors for retinol or vitamin A in the human body [18]. Cowpea is also a source of carotenoids [15,25]. It was reported that the consumption of cowpea leaves improved retinol levels in serum and hemoglobin concentration among preschool children [26]. According to the United States National Institutes of Health, the consumption of one cup (170 g) of boiled cowpea grains can provide up to 66 µg of retinol [27]. Several studies have highlighted the importance of vitamin A in human growth, the immune system, reproduction, and vision [22,28,29].

Carotenoids as pigments also provide distinctive colors (red, orange, and yellow) and some aromas, which make them commercially important compounds in various industries, including health, food, cosmetics, and aesthetic industries [18]. Carotenoids are one of the most widely used antioxidants in the cosmetics industry; they possess antiaging properties and protect the skin against free radicals from solar radiation [30,31]. Hence, the biofortification of carotenoids can serve different market segments and influence consumers’ choices. For instance, the biofortification, extraction, and encapsulation of β-carotene from green sources can play a dual role as a food additive and a substitute to synthetic dies in the food industry [32] and can be used as nutricosmetics in the cosmetics industry [30]. The success of such technologies depends on our understanding of the carotenoid synthesis network and the methods of optimizing and extracting them from specific plant matrices.

## 3. Carotenoid Biosynthesis in Cowpea

**Figure 2:**
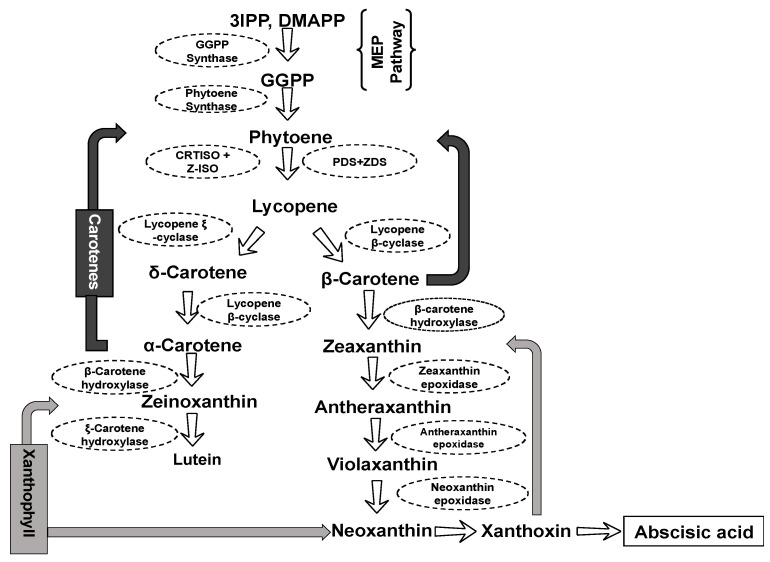
Carotenoid biosynthesis pathway in cowpea. IPP = isopentenyl pyrophosphate, DMAPP = dimethylallyl diphosphate, GGPP = geranylgeranyl diphosphate, PDS = phytoene desaturase (PDS), Z-ISO = z-carotene isomerase, ZDS = z-carotene desaturase, CRTISO = carotenoid isomerase (CRTISO).

Carotenoids are made of polyene hydrocarbon chains consisting of eight isoprene units [33]. The carotenogenesis or biosynthesis of carotenoids is a series of biological reactions with some core sequences conserved across plant species. In cowpea, the first and most determinant step (Figure 2) of carotenogenesis is the condensation of two molecules of geranylgeranyl diphosphate (GGPP, C20) by phytoene synthase (PSY) to form phytoene [5]. Two major enzymes are involved in this step, geranylgeranyl pyrophosphate synthase and phytoene synthase, which are present in all carotenogenic organisms [6]. GGPP originates from the condensation of three molecules (C_5_) of isopentenyl pyrophosphate (IPP) and dimethylallyl diphosphate (DMAPP), a reaction catalyzed by the GGPP synthase [34]. IPP and DMAPP are derivatives of the 2-C-methyl-D-erythritol 4-phosphate (MEP) pathway, which is also involved in the biosynthesis of other important secondary metabolites, such as chlorophylls, Gibberellins, phylloquinone, and tocopherols [35].

The second step in carotenogenesis is a series of desaturation and cyclization reactions, whereby phytoene is converted into hydrocarbon carotenoids (carotenes) and their oxygenated derivatives (xanthophylls) [6]. Phytoene undergoes four sequential desaturations, reactions regulated by phytoene desaturase (PDS), z-carotene isomerase (Z-ISO), z-carotene desaturase (ZDS), carotenoid isomerase (CRTISO), and light-mediated photoisomerization to form lycopene (C_40_H_56_) [36]. Lycopene is then cyclized to produce α- and β-carotenoids through the enzymatic activity of lycopene cyclase [5,36]. This branching point in the carotenoid biosynthesis pathway regulates the ratio of the synthesis of lutein and β-carotene. In one branch, a single enzyme, lycopene β-cyclase (LCYB), introduces a β-ring at both ends of lycopene to form β-carotene. In the other branch leading to lutein formation, β-cyclase and ξ-cyclase introduce one β-ring and one ξ-ring, respectively, into lycopene to form α-carotene [4]. α-Carotene is acted upon by a β-ring hydroxylase to form zeinoxanthin, which is then hydroxylated by an ξ-ring hydroxylase to produce lutein, the major carotenoid present in green tissues such as cowpea leaves [4,37]. β-carotene is hydroxylated in a two-step reaction to zeaxanthin, with β-cryptoxanthin as an intermediate product. Zeaxanthin is converted into violaxanthin, and vice versa, and violaxanthin into neoxanthin, giving rise to abscisic acid [34].

This overview of the carotenoid biosynthesis pathway shows that there is an understanding of the basic process leading to the formation of specific carotenoids in cowpea. The mechanisms involved in the regulation of this process vary among plant tissues [38], elucidating the biochemical network and genetics architecture and controlling the biosynthesis of these compounds in the edible parts of the cowpea.

## 4. Identification and Quantification Methods of Carotenoids in Cowpea

The composition of carotenoids in plants is complex and varies both qualitatively and quantitatively [33] and, thus, requires an accurate method for identification and quantification [39]. Both destructive and non-destructive methods have been developed for the detection and quantification of carotenoids in plants. In this section, we describe the existing methods for carotenoid profiling, highlighting those used in cowpea, and pointed out potential research gaps.

### 4.1. Destructive Methods for Quantification of Carotenoids in Cowpea

Destructive methods are the most widely used techniques in carotenoid analysis. They involve the sampling of the biological plant material followed by specific extraction procedures and their analysis. The choice of extraction method is a very critical factor for achieving a high extraction yield [31]. In cowpea, conventional extraction using an organic solvent, acetone, or a mixture of acetone and hexane, or acetone-hexane and ethanol, at 25–80 °C, is commonly used [25,40,41]. Saini and Keum [31] described a Soxhlet extraction method that uses organic solvents (hexane, ethyl acetate, ethanol, acetone, etc.) at boiling temperature and low pressures as the best conventional method for carotenoid extraction [31,42]. In this process, saponification is sometimes carried out to remove non-targeted compounds such as lipids and chlorophyll [43]; the addition of butylated hydroxytoluene (BHT) helps to prevent the eventual oxidation of the carotenoid compounds.

Other carotenoid extraction methods include supercritical fluid extraction (SFE), which uses a fluid state of carbon dioxide ‘supercritical CO_2_’; on the other hand, some green extraction methods, in contrast, use friendly green solvents from renewable resources of biomass feedstock (e.g., wood, starch, fruits, and vegetable oils) or from petrochemical products that are non-toxic and biodegradable [31]. These methods have not been explored in cowpea yet and merit further investigation along with the assessment of their cost-effectiveness as they are more eco-friendly compared to Soxhlet extraction. However, a thorough evaluation of the different alternatives is required by the experimenter to avoid environmental pollution while minimizing the risks of degradation, auto-oxidation, and isomerization, which can result in bias separation and quantification of the carotenoids from the cowpea samples.

Carotenoid analysis has benefited from advances in various fields, including chemistry, optics, atomic physics, and magnetism, with the development and optimization of spectrometry and chromatography approaches to elucidate the profiles of different carotenoid compounds from biological samples. To date, there are several reviews on the separation and quantification methods of carotenoids in plants and their by-products [44,45,46,47]. Column chromatography is the most widely used technique for carotenoid separation in cowpea [48,49,50]. Column chromatography techniques include classical open-column chromatography (OCC) and high-performance liquid chromatography (HPLC).

Thin-layer chromatography (TLC) is a low-cost and rapid OCC technique used in carotenoid analysis [51]. TLC helps to separate the specific carotenoid compounds (β-carotene and α-carotene; β-cryptoxanthin; and lutein and zeaxanthin) [31,52]. However, there is very scant information on the use of TLC for carotenoid analysis in cowpea [49], and even in that case, it was used in combination with HPLC. In fact, the low resolution of TLC often limits its large application prospects [39,53].

In contrast to TLC, liquid chromatography (LC) helps to achieve both the separation and quantification of specific carotenoids and their isomers. HPLC analyses using C18 and C30 columns as stationary phases have successfully been deployed for carotenoid analysis in cowpea [25,41,54,55]. Ultra-performance liquid chromatography (UPLC) and ultra-high-performance liquid chromatography (UHPLC) are modern LC techniques used for the separation of carotenoids in cowpea [40]. The later techniques operate at higher pressures (≥15,000 si) and possess high selectivity compared to HPLC (max < 6000 psi), which increases the speed and resolution of the analysis when coupled with the C30 column [39,56]. This technique was also deployed to assess carotenoids in cowpea [40,57].

The use of multiple techniques in a single platform has become the approach of choice for the separation and quantitative analysis of carotenoids as they are more effective. Routinely, LC is combined with spectrophotometry techniques to increase the precision of the analytical procedure [49,51]. Most carotenoids absorb light in the range of 400–500 nm [46]; hence, ultraviolet-visible (UV-VIS) spectrophotometry is used with LC to quantify carotenoid contents in the plant extracts [44,45,47]. LC-UV-VIS is the common platform used for carotenoid analysis in cowpea [25,41,58]. UV-VIS may fall short to clearly separate all carotenoids, especially the trans/cis isomer forms [44,46]. So, the use of mass spectrophotometry (MS) helps to overcome some of these limitations in the traditional UV-VIS technique [40,57]. MS relies on the power of ionization techniques to transform the liquid or solid phase of the analytical sample into an ionized gas phase and the separation of carotenoid compounds through the measurement of their mass-to-charge ratio of ions [59,60]. Atmospheric pressure chemical ionization (APCI) is the most commonly used ionization technique in the LC/MS analysis of carotenoids in cowpea [49,61]. Other ionization techniques include electron impact (EI), fast atom bombardment (FAB), matrix-assisted laser desorption/ionization (MALDI), electrospray (ESI), pressure photoionization (APPI), and atmospheric pressure solid analysis probe (ASAP) [59,60]; however, there is no evidence of their use in carotenoid profiling in cowpea.

### 4.2. Non-Destructive Analysis of Carotenoid Content in Cowpea

The development of non-destructive methods is proposed as a quick alternative to the destructive methods for timely and on-farm/field assessments of carotenoids in plants [62]. The absorption of carotenoids in the visible range makes it possible to detect and quantify carotenoids through microscopy and/or spectroscopy [63]. For instance, a combination of light microscopy, UV-Vis transmission spectroscopy, and diffuse reflectance spectroscopy is used for the characterization of carotenoids in tomato, carrot, and gac fruit [64]. Near-infrared reflectance spectroscopy (NIRS) is the most widespread reflectance spectroscopy currently in use. NIRS was successfully deployed for assessment in maize [65], cassava [66], and sweet potato [67]. While the non-destructive assessment of carotenoids is still a relatively new approach, it has not been introduced in cowpea, suggesting there is an avenue for technology development in cowpea research.

## 5. Determinants of Carotenoid Biosynthesis and Accumulation in Cowpea

Profiles of different metabolites in plants, especially the secondary metabolites, are the results of a continuous balance between intrinsic characteristics and exogenous factors controlling plant growth and development. Hence, changes in only one single factor may induce significant fluctuations in plant metabolites. Verma and Shukla [68] identified four groups of factors, namely genetic, ontogenic (growth and development), morphological, and environmental factors, which can influence the production of secondary metabolites in plants. In this section, we described the possible effects of these groups of factors on the profiles of carotenoids in cowpea.

### 5.1. Genetic, Ontogenic, and Morphological Basis of Carotenoid Variation in Cowpea

There is evidence of the natural accumulation of carotenoids in cowpea, and the nature and concentration of these compounds vary among genotypes, organs, and growth and developmental stages (Table 1). Previous studies on carotenoid analysis in cowpea revealed there is a variation in total carotenoids (0 to 9.46 µg/g) in the dry grains [15,25,58,69,70].

Growth and development also influence the carotenoid content. Carotenoids and chlorophylls are two important components of photosystem (PSI and PSII) units of protein complexes involved in the primary photochemistry of photosynthesis [71]; hence, their concentrations can vary with plant growth. It was observed in the wild Fabaceae species that during germination, the total content of photosynthetic pigments increased in parallel to changes in the relative abundance of carotenoids [72]. Similarly, germination also induces significant changes in carotenoid content in cowpea. Total carotenoid content varied from 16.7 µg/g [58] to 122.88 ug/g [41] in 2- and 15-day-old cowpea sprouts, respectively. Luthria et al. [73] observed an increase in β-carotene from 0.13 ± 0.05 μg/g in the dry cowpea grains to 0.19 ± 0.3 μg/g in 2-day-old sprouts. Elsewhere, variation from 1.8 to 29.4 µg/g of carotenoid content was reported among fresh pods of 37 cowpea accessions [74], suggesting that the biofortification and promotion of the consumption of fresh cowpea pods can also be envisioned as a strategy for food diversification and micronutrient deficiency alleviation. The highest variation in total carotenoids was found in cowpea leaves from 0.44 to 2245 µg/g [40,54,69,70,75].

In terms of specific carotenoids, lutein is the most abundant present in cowpea [7,40,41,55]. The reported values of lutein content in cowpea range from 0 to 0.49 µg/g in the seeds [58] to 1246 µg/g in leaves of adult plants [40]. Similar trends were observed in the variation in β-carotene between dry grains (0–0.1 µg/g) and leaves (184.5–958 µg/g) of cowpea [40,58]. Recent studies have indicated significant variation in carotenoids among five-day-old sprouts of a cowpea diversity panel, with up to 1824 µg/g lutein accumulated in the most carotenoid-rich sprouts, further supporting the benefits of using sprouts in food fortification programs [55]. In most cases, the lutein content is approximately two- to three-fold the concentration of β-carotene in the different cowpea organs (Table 1).

Studies on carotenoid biosynthesis in soybean showed different patterns of carotene and xanthophyll accumulation among yellow, black, and green seed-coated soybean [76]. Significant variation was also observed in seed coat color in cowpea [77], which was reported to influence the variation in secondary metabolites, including phenolics, flavonoids, and anthocyanin [78,79]. Therefore, the study of the variation in carotenoid contents among different cowpea morphological groups, especially seed coat color groups, can guide the selection of a germplasm to start a breeding program for biofortified cowpea varieties.

**Table 1:** Methods of quantification and variation in carotenoid content in cowpea.

Plant Organs	Quantification Methods	Total Carotenoids	α Carotene	β Carotene	Lutein	Zeaxanthin	Cryptoxanthin	Authors
		µg/g						
Seeds	Spectrophotometry	-	-	0.1	-	-		[73]
	HPLC _(C30 Column)_	9.46	-	-	4.3	5.5	-	[15]
	HPLC _(C18 Column)_	0.6	-	-	0.6	0	-	[25]
		0.95	-	0.04	0.9	<0.01	-	[69]
		0.6	0	0.06	0.5	-	0.03	[58]
Leaves	Spectrophotometry	436.8	-	-	-	-	-	[70]
	HPLC _(C18 Column)_	-	-	806.0	-	-	-	[26]
	HPLC _(C30 Column)_	570	7.2	184.5	360	18.6	3.3	[54]
	UHPLC _(C30 Column)_	2245	-	958	1246	10	-	[40]
Sprouts	Spectrophotometry	-	-	0.2	-	-	-	[73]
	HPLC _(C18 Column)_	16.7	2.1	2.8	2.5	-	0.17	[58]
	HPLC _(C30 Column)_	253.7	5.9	66	162.1	-	-	[41]
		-	-	652	1824	393	-	[55]

### 5.2. Exogenous Factors Influencing Carotenoid Biosynthesis in Cowpea

- *Light exposure and intensity*

Carotenoids absorb light in a broader range of wavelengths in the blue region of the visible-light spectrum and subsequently transfer the energy to chlorophyll [18]. Light and circadian oscillations during plant growth can alter the expression profiles of different genes involved in carotenoid biosynthesis [36]. Light and circadian oscillations were reported to influence the availability of isoprenoid isomers (IPP and DMAPP), which are upstream precursors of carotenoids in cowpea [18,36]. The exposition of cowpea seedlings to different light-emitting diodes influenced seedlings’ growth, with significant changes in the patterns of carotenoid compounds [41,80]. The duration and intensity of light exposure are important determinants of fluctuations in carotenoid contents. However, high light intensity could be a limiting factor in carotenoid biosynthesis. An increase of about 0.4-fold of carotenoid content was observed in the leaves of cowpea grown under low-light conditions, as compared to sunlight-grown cowpea [81].

- *Temperature*

Temperature is an important environmental factor that influences plant growth and development. An increase in ambient temperature affects the physiology, biochemistry, and regulation pathways [82]. The effect of an increase in temperature on carotenoids may vary among plant genotypes and species. Lefsrud et al. [83] reported a contrasting effect of ambient temperature increases on lutein and β-carotene content in kale and spinach. In cowpea, an increase in ambient temperature (38/30 °C; day /night) showed a positive effect on carotenoid content in the leaves [84]. Cowpea is more tolerant to temperature and may thrive under a large range of heat waves, as compared to other grain legume crops, such as *Phaseolus vulgaris*, *Vicia faba*, and *Pisum sativum* [85]. However, temperatures beyond 40 °C may cause significant damage to the plants, especially during the reproductive stage [86,87]. A significant decrease of ~40% in photosynthetic pigments was observed in the wild relative of cowpea (*Vigna radiata* L.) as a result of an increased (>40/25 °C) day and night temperature [88]. Hence, the assessment of the critical temperature that specific genotypes may withstand will help to act upon this factor efficiently for the optimum accumulation of carotenoids in cowpea.

- *Plant nutrition and carotenoid biosynthesis*

Nutrients are indispensable for biochemical reactions and the production of photosynthates in plants [89]. Plant nutrients are grouped into major nutrients (e.g., nitrogen, carbon, phosphorus, potassium) and minor nutrients (e.g., copper, zinc, iron, manganese); the balance between them supports plant growth as well as resistance to diverse stresses. Hence, the choice of nutrition type or plant growth media can induce changes in carotenoid biosynthesis in plants. For instance, the treatment of cowpea plants with elevated atmospheric CO_2_ (360 and 720 Umol·mol^−1^) increased the carotenoid content in the leaves [84]. It was reported that the application of inorganic fertilizer (100 kg urea+300 kg single super-phosphate ha^−1^) resulted in 0.36 mg·g^−1^ increase in carotenoid content in cowpea pods, as compared to organic manure-treated plants [90].

Minor plant nutrients, on the other hand, are required in small quantities. They are often supplied to the plant in the form of salt; hence, a surplus may be detrimental. In fact, salt stress can decrease the expression level of genes involved in the carotenoid biosynthesis pathway, resulting in a low carotenoid content in the plant [91,92], (Table 2). Salt stress, especially a high concentration of salt (50–200 mM), has been reported to delay the growth of cowpea seedlings, reducing both the carotenoid content and net photosynthetic rate [93]. Furthermore, the elicitation of broad bean sprouts with a high concentration (240 and 300 mM) of salt (NaCl) reduced the carotenoid content [92,94]. Similarly, the treatments of mungbean seedlings (*Vigna radiata*) with different concentrations of sodium chloride (200 and 250 mM) [95] or manganese sulphate (0.1 to 5 mM) considerably decreased the carotenoid content, as compared to the untreated seedlings [96]. Therefore, plant nutrients, especially salt as a stress factor, greatly influence the build-up of carotenoid in plants, and this is dose and species dependent. Such changes in the content of carotenoids in cowpea due to nutrient uptake may be the result of changes in the expression profiles of the genes involved in the biosynthesis pathway. For instance, it was reported that the treatment of the watermelon plant with salt solution significantly reduces the carotenoid content through the downregulation of the expression level of i phytoene synthase (PSY), phytoene desaturase (PDS), zeta carotene desaturase (ZDS), and lycopene beta cyclase (LCY-β) [91]. Such studies are scant in cowpea, meaning there is a need to deploy research efforts to uncover the effects of salt treatment on the genes involved in carotenoid biosynthesis and to establish the optimal nutrition system for inducing a positive change in carotenoid content in cowpea, concurrently with efforts to improve all other physiological and biological functions.

- *Plant hormones and carotenoids*

Plant hormones are key components of biological and physiological processes in plants [97]. They regulate the biosynthesis of metabolites including carotenoids in response to the intrinsic factors and/or exogenous factors influencing plants’ growth and development [98,99]. For instance, in cowpea, an increase in carotenoid content was observed in plants treated with an exogenous application of salicylic acid as evidence of systematic acquired resistance against diseases [100].

Carotenoids, particularly β-carotene, are precursors for two important hormones in plants, namely strigolactones and abscisic acid [5]. Strigolactones and abscisic acid regulate plant development and interaction with the environment [101]. An increase in ABA biosynthesis was found in 8-day-old cowpea seedlings under drought stress as a result of an increase in the expression level of NCED (9-cisepoxycarotenoid dioxygenase), a gene that increases the accumulation of lycopene and β-carotene, the upstream compounds in the ABA biosynthetic pathway [102,103].

**Table 2:** Effects of elicitation on carotenoid accumulation in cowpea and other related legume grains.

Elicitors	Crops	Growth Stage	Treatments	Treatment Duration	Effects	Authors
NaCl	Cowpea	1-week-old seedlings	60–200 mM	14 Days	Reduce total carotenoids	[93]
	Broad Bean	6-week-old seedlings	60–240 mM	10 Days	Reduce total carotenoids	[94]
	Common bean	3-week-old seedlings	50–200 mM	7 Days	Reduce total carotenoids	[104]
	Mungbean	1-week-old seedlings	200–250 mM	14 Days	Reduce total carotenoids	[95]
UVB	Cowpea	Germinated seeds	470 nm	14 Days	Increase profiles of all carotenoids	[41]
Fluorescence	Mungbean	Germinated seeds	400–700 nm	5 Days	Increase total carotenoid content	[105]
Dark	Mungbean Soybean	Germinated seeds	Dark conditions	5 Days	No positive effect on carotenoids compared to light treatment	[105,106]

## 6. Integrated Approach for Carotenoid Biofortification in Cowpea

### 6.1. Breeding for Increased Carotenoid Content in Cowpea

**Figure 3:**
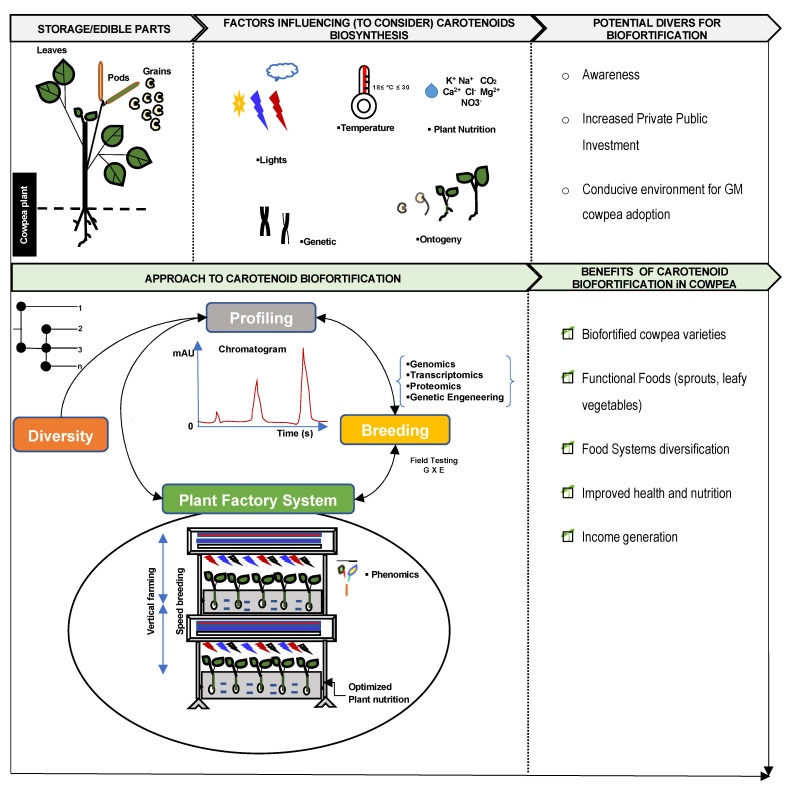
Road map for biofortification of carotenoids in cowpea.

In recent decades, biofortification has gained importance as one of the most sustainable ways to supply micronutrient-rich foods for alleviating hidden hunger and malnutrition worldwide [107,108]. In regard to carotenoids, most of the research efforts have focused on increasing the carotenoid precursors of vitamin A (provitamin-A carotenoids), such as β-carotene, α-carotene, and β-cryptoxanthin. Consequently, there have been significant advances in breeding for provitamin-A carotenoid varieties in some major crops, including maize, cassava, and sweet potato [8,109]; however, the potential of the legume grain crops including cowpea is still untapped. It is, thus, important to leverage the lessons and progress in other crops, in other to define an effective approach (Figure 3) for the biofortification of carotenoids in cowpea.

The extent of genetic gain in breeding cowpea for enhanced carotenoid content depends on the knowledge of the genetic diversity for the trait. The screening and evaluation of crop diversity is the first and most important step in breeding for carotenoid-biofortified varieties [110]. The literature survey showed that information on the diversity of carotenoid content in cowpea is still very scant. Nonetheless, there have been extensive efforts on the collection and conservation of cowpea genetic resources, with large germplasm collections maintained at different genes banks, which can be used as working materials: IITA (15,003 accessions), the United States Department of Agriculture (USDA)–Genetic Resources Information Network (7737 accessions), and the University of California, Riverside (UCR), collections of 6000 accessions [111,112]. To save time and resources, the screening and evaluation can be narrowed down to the established mini-core collections from these various gene banks, which capture most of the existing diversity in the crop. The mini-core collections include 298 accessions from the IITA collections [112]; 368 accessions from the UCR collections [113]; and 369 accessions from the USDA cowpea germplasm [114].

The assessment of the genetic diversity for carotenoid content should integrate both biochemical profiling and molecular analyses. There are known genes, such as the phytoene synthase (PSY1), β-carotene hydroxylase (CHYB), lycopene β, and ξ cyclase (LYCB and LYCE), that play significant roles in the biosynthesis of carotenoids in plants [115,116]. Hence, the screening of the cowpea diversity panels targeting these genes can reduce the cost and time needed for profiling and help to precisely identify accessions with the trait of interest.

Genomics interventions for important and quantitative traits such as carotenoid content can begin at the early stage of the breeding scheme by tapping into the genetic and genomics resources of the crop [11,110]. The advances in cowpea genomics enabled the development of a reference genome [117], genetic linkage maps [118,119,120,121], and diverse molecular markers and marker systems (RFLP, SNPs, SSRs, KAPs, etc.) [122,123,124] to support the development of improved cowpea varieties. The assessment of genetic diversity for carotenoid content in cowpea can, therefore, be conducted along with screening and validation of the existing markers and their possible association with those known genes involved in carotenoid biosynthesis to identify quantitative trait loci amenable to the smooth implementation of marker-assisted selection (MAS) for enhanced carotenoid content in cowpea.

To anticipate the low genetic diversity reported in cowpea [112], deploying mutagenesis [125] will help to broaden the diversity in the crop. For this purpose, the use of TILLING (Targeting Induced Local Lesions In Genomes), a technique that combines chemical mutagenesis and high-throughput screening of SNPs by mismatch detection [126], will help to achieve fast progress in broadening the genetic basis and improving the carotenoid content in cowpea.

Once the working germplasm with the elite or potential genotypes is identified, the next step involves embracing hybridization between accessions. Notably, at this stage, the objective consists of conducting smart combinations among genotypes using appropriate mating design (Diallel, North Carolina Mating Design) and population development techniques (Single Seed descent and Backcross) [127,128], which will help to estimate the variance components, gene actions as well as heritability [127] in order to dissect the genetic architecture of carotenoid biosynthesis in cowpea to support the breeding scheme. Previous research indicated the predominance of additive gene effects over the effects of non-additive genes in the inheritance of carotenoid content in plants [129,130], suggesting that the use of the proposed designs can also facilitate introgression of the trait into elite and farmers’ preferred cowpea cultivars. Finally, the evaluation of the different genotypes and crosses developed across environments will enable us to account for the effect of the interaction of genotype and environment on the profiles of the different carotenoids, enabling the preliminary and advanced yield trials along with participatory evaluations of the superior genotypes to increase their adoption for different end-users.

### 6.2. Harnessing the Power of Plant Factory System, Speed Breeding, and Omics

Facing the increasing demand for quantity and quality foods to feed an ever-growing population, there has been a steady shift from traditional rain-fed agriculture to indoor growing systems/vertical farming. The evolution of this approach has given rise to the plant factory system, which is referred to as a closed plant production system in which ventilation is kept at a minimum, and artificial light is used as the sole light source for plant growth [131]. The adoption of this production practice has been very fast in horticultural crops. Nowadays, vegetables, such as spinach, tomato, and kale, are produced in factory systems [132,133,134], which have been customized to meet specific market demands including high-phytochemical and nutrient-dense products. This system can now be extended to agricultural crops with the recent development of the speed breeding method [135].

Speed breeding is a customized plant factory system for field crops in fully enclosed, controlled-environment growth chambers, which enables the production of many generations, up to six generations of crops per year [135]. Speed breeding shortens the growth cycle and the time needed for developing new crop varieties. The technology was first implemented for long-day crops such as wheat and canola [135] and has recently been extended to short-day crops [136], suggesting that the system can be optimized for cowpea.

As highlighted earlier, light is one of the factors influencing carotenoid biosynthesis in cowpea. The manipulation of light signaling can help to alter the color and nutritional value in plants, resulting in the production of novel functional foods [20]. Therefore, the optimization of the speed breeding technology for cowpea can help to control the lighting characteristics (intensity and duration) for increased carotenoid contents in the edible part of the plants, including sprouts, leaves. and green pods, to be used as functional foods (Figure 3). This growth system also offers the flexibility of a choice of plant-growing substrate (e.g., rockwool, top soil) to monitor the nutrition of the plants and to apply appropriate chemical elicitors (Figure 3) that have a positive impact on carotenoid biosynthesis in plants [100,137].

Furthermore, the implementation of this approach in cowpea can also take advantage of the advances in the field of phenomics to deploy non-destructive tools for carotenoid detection and quantification [64,138,139] in cowpea. This will help to minimize the cost of extensive profiling and also generate quality phenotypic data [140] to guide our understanding of the physiological and genetic basis of carotenoid biosynthesis in cowpea. Notably, the metabolomics regulation network of carotenoid biosynthesis in cowpea is still not fully documented. The development of the metabolomic database of carotenoids in cowpea and its combination with genomics, transcriptomics, and proteomics [39,140,141], coupled with the plant factory system, can help shorten the breeding cycle and broaden our understanding of the biology of carotenoid accumulation and its optimization in cowpea sprouts, microgreens, leaves, and grains.

### 6.3. Genetic Engineering for Increasing Carotenoid Content in Cowpea

Genetic engineering has emerged as a technology to overcome the slow process of conventional breeding and the lack of diversity for carotenoid traits among plant germplasm [142]. A recent report [25] on the side effects of domestication on the nutritional quality of legume crops in the Fabaceae family highlighted the decline in the contents of carotenoids following the domestication process. According to these authors, there was a decrease in carotenoid content (0.6 ± 0.1 μg/g) in the cultivated cowpea, about three-fold of the content in the wild cowpea (2.3 ± 0.5 μg/g) due to domestication. The wild cowpea gene pool may, therefore, be a source of favorable genes for increased carotenoid content in cowpea

To date, there is no evidence of genetically modified (GM) biofortified legume grains or pulses [143]. A comparative study of the mechanisms controlling the biosynthesis in the wild and cultivated cowpea plant will provide an avenue to perform guided mutations in the cultivated cowpea genome. The CRISPR/Cas9 (Clustered Regularly Interspaced Short Palindromic Repeats/Cas9 protein) gene-editing technology can assist in precisely conducting the target mutation [142,144,145]. A similar approach was adopted in sweet potato with an increase of 4- to 130-fold of zeaxanthin content in the transgenic potato [146]. On the other hand, interspecific genes transfer between the wild cowpea and the cultivated cowpea genome through genetic transformation [147,148,149]. In addition, the donor organisms could be from closely related species in the Fabaceae family and plant or animal species with the genes of interest. For instance, metabolic engineering of the phytoene synthase gene (crtB) from bacteria (*Erwinia uredovora*) helped to achieve a 150-fold increase in β-carotene in transgenic eggplant callus [148]. The duplication of these approaches in cowpea can also help increase the carotenoid content. However, the environmental and health concerns about genetic engineering and its products globally, and especially in many regions where cowpea is a staple food crop, seem to portray genetic engineering as an avenue of last resort. Nonetheless, some tangible progress has been made with the adoption of Bt cowpea in Nigeria [150], the leading cowpea producer in the world, indicating a promising future to escalate this technique for increasing carotenoids in cowpea. Hence, continuous public awareness raising and increasing advocates in the private and public partnerships to scale up the research technologies in developing countries will be a strong levier in the successful deployment of genetic engineering for micronutrients including carotenoid contents in cowpea (Figure 3).

## 7. Conclusions

This review emphasized that carotenoids are important micronutrients for human health and nutrition. Increasing these micronutrients in cowpea will have a range of applications in the health and food industries. Though the pathway of carotenoid biosynthesis in cowpea encompasses some core steps conserved across higher plants, there is still a need to elucidate the biochemical network and genetic architecture controlling its biosynthesis and accumulation in cowpea. There was evidence of variation in the carotenoid profiles among genotypes, organs, and growth and developmental stages in cowpea; however, the data on the genetic diversity of the trait in cowpea are very scant. This suggests the extensive investigation of the natural variation in carotenoids and the deployment of strategies (mutagenesis and genetic engineering) to increase the genetic basis of this trait in cowpea. Furthermore, the variation in the profiles of carotenoids in cowpea is influenced by exogenous physical and chemical factors, such as light intensity and duration, plant nutrition, and temperature, which induce physiological changes, resulting in fluctuations in the carotenoid content in edible storage organs. It was established that the manipulation of these factors in an integrated system can lead to a significant increase in carotenoids in cowpea. The proposed system is set to harness the power of omics coupled with speed breeding and genetic engineering, drawing from the lessons in other crops to achieve significant genetic gains and increases in the carotenoid content in cowpea. The approach described herein is transferable to other pulses crops, the potential of which is still underexploited for a food-secure planet.

The information presented in this review did not cover factors such as bioavailability (bioaccesibility and bioactivity) and losses of carotenoids during processing and cooking, which are equally important to assess along with the research on biofortification to make them fully accessible for human health and nutrition.

## Data Availability

No new data were created in this study, and references were given in the text wherever data were obtained from other sources.
